# Repairing Vulcanite Work

**Published:** 1885-01

**Authors:** S. B. Palmer

**Affiliations:** Syracuse, N. Y.


### REPAIRING VULCANITE WORK.

BY DR. S. B. PALMER, SYRACUSE, N. Y.

Scarcely any two workmen conduct the operation of repairing broken or cracked dentures alike. This is of little consequence, provided the work be well done, except, perhaps, in the appearance and saving of time. It is not my intention to instruct those whose experience and practice in that line far exceeds my own, nor to consume time in giving methods of flasking, finishing etc.

We often see cases greatly disfigured by under-cuts, dovetails, and other devices, which were intended to secure union between the old and new material, a process as unnecessary as it would be in the use of glue in cabinet work. This is no theory, but a fact well substantiated by a trial of seventeen years. Observe the conditions, which are few and simple, and it will be seen that separation of the parts on the line of contact is impossible. There may be a score of methods that will accomplish the same object. The following certainly will:

Prepare the surfaces with file, bur, or scraper, as most convenient. Bevel to a thin edge if possible. When the parts are ready to receive the wax for building up, paint the surfaces with a solution of chloroform and such rubber as the case is made of, red or black. This coating should be thin, so that it may only show a gloss when dry; not a covering of rubber, as would be the case were it thick as cream. Under no circumstances attempt to fill thin spaces or out-of-the-way places with rubber softened by a solvent. Such preparations are brittle and porous. After the surfaces have been once varnished, wax or oil does not injure or prevent union when boiling water is used to remove the same. Portions like the rim and clasps may be supplied with strips of rubber instead of wax, covering them with plaster before flasking. By leaving space in packing for the rubber to reach that which is imbedded, there will always be perfect union; an effectual method of removing wax consists in boiling the case in water ; a pint basin of water heated over a lamp is sufficient. By the use of a four ounce metal syringe, every particle of wax may be driven out and made to float upon the surface of the water. A piece of paper drawn over the water takes up the wax, and the case comes out clean, and with a surface well prepared to receive the rubber used in packing. Other methods may answer equally well, but the above will give positive success.

